# Are Leukocyte and Platelet Abnormalities and Complete Blood Count Ratios Potential Prognostic Markers in Canine Sepsis?

**DOI:** 10.3389/fvets.2020.578846

**Published:** 2020-10-30

**Authors:** Alessio Pierini, Eleonora Gori, Ilaria Lippi, George Lubas, Veronica Marchetti

**Affiliations:** Department of Veterinary Sciences, Veterinary Teaching Hospital “Mario Modenato”, University of Pisa, Pisa, Italy

**Keywords:** white blood cells, inflammation, dog, prognosis, septic

## Abstract

**Background:** Sepsis is a common disease in which early diagnosis and prognosis assessment are the main aims in order to arrange a prompt and effective treatment.

**Objectives:** (1) To compare leukogram parameters (WBC, segmented and band neutrophils, lymphocytes, monocytes), platelet count (PLT), mean platelet volume (MPV), and some leukocyte/platelet ratio such as NLR, NBNLR, PLR, and MLR between dogs with systemic inflammatory response syndrome (SIRS) and sepsis. (2) To investigate any difference in the trend of these latter parameters between survivors and non-survivors septic dogs.

**Animals:** 57 dogs with confirmed sepsis and 57 dogs with non-septic SIRS.

**Methods:** A review of the medical records was conducted in order to find dogs with sepsis. Sepsis was defined as the presence of an infectious focus with fulfillment of systemic inflammatory response syndrome criteria (SIRS). Septic dogs had to have a CBC at admission and another CBC within 48 h from the previous timepoint. Purebreds with CBC breed-related abnormalities were excluded, together with dogs without confirmed sepsis and dogs with only a single CBC. NLR, NBNLR, PLR, and MLR were calculated. Univariate analysis of all blood parameters studied was assessed between SIRS and septic dogs. Generalized Estimating Equations models for repeated measures were used to test if the blood parameters studied were modified between survivors and non-survivors in the septic group.

**Results:** Septic dogs had lower median segmented neutrophils count and NLR compared to SIRS dogs (*p* = 0.02 and *p* = 0.04, respectively). Lastly, septic dogs had a higher prevalence of toxic neutrophil than SIRS dogs (*p* = 0.01). We found that for a 1-unit increase of PLR and MLR, the risk of death increased by 50.5 and 60%, respectively.

**Conclusion and Clinical Importance:** Evaluation of NLR at hospital admission may be a useful marker of inflammation, although it showed low sensitivity in differentiating SIRS and septic dogs. The monitoring of some CBC parameters, especially PLR and MLR may be useful in the establishment of prognosis in septic dogs.

## Introduction

Sepsis is a common disease and an important cause of morbidity and mortality in both humans and dogs (1, 2). An early diagnosis of this syndrome and establishment of patients' prognosis are still the main aims in order to set up a prompt and effective treatment (1, 3). In dogs, the potential diagnostic and prognostic value of leukocyte and platelets parameters has been recently investigated in few studies (4–8). The first two studies investigated the prognostic and diagnostic utility of a degenerative left shift in dogs with various diseases and the delta neutrophil index in septic dogs with some discrepancies (4, 5). Burton et al. concluded that the presence of a degenerative left shift was associated with an increased mortality (4), although other authors failed to find an association between degenerative left shift and mortality in septic dogs (5). To the best of our knowledge, platelet abnormalities were taken into account in septic dogs only by Llewellyn et al. (6). In this study, the authors highlighted that dogs with septic peritonitis have increased frequency of thrombocytosis or thrombocytopenia with increased MPV, plateletcrit, and platelet distribution width and an increased mean platelet volume (MPV) that might be used as indicator of increased risk of mortality in dogs treated with surgery (6). In Hodgson et al. (7), dogs with septic peritonitis had significantly higher WBC, segmented and band neutrophils count than SIRS dogs (7).

In these latter years, since the difficulties in the differentiation between sepsis from systemic inflammatory response syndrome (SIRS) based on leukocytes and leukocytes differential count and eventually platelets, several CBC indexes have been investigated. In human medicine, the neutrophil-to-lymphocyte ratio (NLR) has been found as an independent predictor of morbidity and mortality in several clinical situations including sepsis (9–11). In veterinary medicine, NLR and platelet-to-lymphocyte ratio (PLR) has been investigated in SIRS and sepsis (7, 8). Firstly, NLR and PLR were compared between healthy dogs and dogs with periodontitis and oral tumors, concluding that dogs with oral tumors had higher NLR and PLR compared to healthy ones (12).

Subsequently, one study failed to find a significant NLR difference between dogs with or without sepsis and between survivors and non-survivors (7). NLR and band neutrophil-to-neutrophil-to-lymphocyte ratio (NBNLR) were investigated in dogs with SIRS and sepsis. NLR resulted significantly lower in septic dogs compared to SIRS dogs, although no significant association between NBNLR and mortality was found (8). The monocyte-to-lymphocyte ratio (MLR) is another CBC index, which has been proved to be prognostic in human cancer, rheumatic diseases and in *Klebsiellae pneumonia* infection (13–15).

We hypothesized that dogs with sepsis may have some leukocyte or platelets abnormalities that may be able to help to differentiate dogs without sepsis and with systemic inflammatory response syndrome (SIRS). The first aim was to compare leukogram parameters (WBC, segmented and band neutrophils, lymphocytes, monocytes), platelet count (PLT) and MPV, and leukocyte/platelet (WBC/PLT) ratios such as NLR, NBNLR, PLR, and MLR between SIRS and septic dogs. Since we hypothesized that non-survivor septic dogs may have a different behavior in leukocyte, platelet and WBC/PLT ratios were compared to survivors during hospitalization. Our secondary aim was to investigate any difference in the trend of these latter parameters between survivors and non-survivors septic dogs.

## Materials and Methods

### Medical Records Review and Case Selection

Medical records from University of Pisa Veterinary Teaching Hospital were electronically searched for dogs with potential sepsis using the terms “sepsis,” “septic inflammation in cytology,” “abscess,” and/or “positive bacterial culture” from 2014 to 2020. Records containing one of these words were reviewed to confirm the presence of sepsis. Sepsis was defined as the presence of an infectious focus with fulfillment of systemic inflammatory response syndrome criteria. Systemic inflammatory response syndrome (SIRS) was defined if there were at least two of the following parameters: rectal temperature <38.1 or >39.2°C, heart rate >120/min, respiratory rate >20/min, and total WBC count >16 × 10^9^/L, <6 × 10^9^/L or band neutrophils >3% of the WBC count (8, 16). To be included in the final cohort, septic dogs should have a complete blood count (CBC) at hospital admission (T0) and another CBC within 48 h from the previous timepoint (T1). Each CBC was performed and as a part of routine care/monitoring of septic dogs using a laser cell counter^1^ and a blood smear stained with May-Grunwald Giemsa^2^ examined microscopically or reviewed by a single clinical pathologist Dip. ECVIM-CA, with more than 20 years of experience (G.L). The leukogram differential count (WBC, neutrophils, band neutrophils, lymphocytes, monocytes), PLT, MPV, and the presence of toxic neutrophils were recorded for each dog. Neutrophilic toxicity, which is routinely evaluated microscopically by a trained clinical pathologist, included: increased cytoplasmic basophilia, cytoplasmic vacuolation, presence of Döhle bodies, and toxic granulation (17). For PLT evaluation, both the PLT count and estimation were taken into account and only dogs with concordance between these two parameters were included.

Dogs were excluded if sepsis was not confirmed from the review of medical records and if information regarding treatment, and outcome were not clearly based on the evaluation of the medical records. In addition, purebreds with known breed-related hematologic features, as Cavalier King Charles Spaniels, Asian breeds (Akita Inu, Shiba Inu.) and Greyhounds, were excluded to avoid the interpretations biases of their platelet count and characteristics.

For the first aim a group of 57 SIRS dogs was included. The SIRS group included dogs taken from our previous study (8). The SIRS group had to have at least two of four SIRS criteria without any septic focus. The CBC admission parameters of interest of SIRS dogs were recorded. The absolute values of the leukocyte used for the calculation of the different WBC/PLT ratios were derived from the differential count collected from the well-stained smear evaluation carried out by an experienced and trained clinical pathologist. The NLR was calculated as absolute segmented neutrophils + band neutrophils divided by absolute lymphocytes (7, 8). The NBNLR was calculated as band neutrophils divided by segmented neutrophils divided by absolute lymphocytes (8). The PLR and the MLR were calculated as the ratios of PLT and monocytes absolute counts to lymphocytes, respectively. From the electronic medical records database, data regarding signalment information (age, sex, and breed), final diagnosis, and potential comorbidities were collected for both SIRS and sepsis groups. Survival information and the focus of sepsis were obtained from the medical records. Mortality rate was evaluated at hospital discharge and dogs were divided in non-survivors (died during hospitalization) and survivors (survived to the discharge).

### Statistical Analysis

Graphical and statistical analysis were performed using commercial software^3^^,^^4^. All the continuous parameters (age, WBC, segmented neutrophils, band neutrophils, lymphocytes, monocytes, PLT, MPV, NLR, NBNLR, PLR, and MLR) were tested for normality distribution using Kolmogorov-Smirnov test. Normally distributed variables were reported as mean ± standard deviation, meanwhile non-normally distributed data were reported as median and 25th−75th percentile. For the first aim, a univariate analysis of leukogram parameters (WBC, segmented neutrophils, band neutrophils, lymphocytes, monocytes), platelet count, and mean platelet volume (MPV), and NLR, NBNLR, PLR, and MLR between SIRS and septic dogs was performed using Mann-Whitney *U*-test since all the continuous variables were non-normally distributed. For all hematologic parameters taken into account, a receiver operating characteristic (ROC) analysis was performed and the area under the curve (AUC) was calculated. Diagnostic cut-offs and their sensitivity and specificity were determined according to the maximum Youden index. Generalized Estimating Equations (GEE) models with robust standard errors for repeated measures were used to test if the changes in leukogram and platelets parameters and WBC/PLT ratios were significantly different between survivors and non-survivors in septic group. In every GEE model, each dog ID was used as subject variable and time as within-subject variable. A GEE binary logistic regression model was used and the outcome (survivor/non-survivor) was chosen as a dependent variable. In each model, time and each parameter (WBC, segmented neutrophils, band neutrophils, lymphocytes, monocytes, PLT, MPV, NLR, NBNLR, PLR, and MLR) were set as predictors, as fixed factor and covariates. In each model, main effects and interaction of factors and covariates were tested. The best correlation structure was selected based on the quasi-likelihood information criteria (QIC). The model with the smallest QIC value was chosen as the most parsimonious model with the best correlation structure. Lastly, a Chi-square test was used to investigate if the focus of sepsis was associated with the mortality. A *p* < 0.05 was considered statistically significant for all the calculations.

## Results

### Study Population

An initial population of 285 dogs was initially screened for eligibility. Eighty-four dogs were excluded since, based on medical records review, they did not satisfy sepsis criteria. Seventy-nine dogs had the T1 CBC performed more than 48 h from the T0. Sixty-four dogs were excluded as they were lacking of the T1 CBC and, lastly, only one dog was excluded because it was belonging to Cavalier King Charles Spaniel breed. Thus, the final cohort of septic dogs was composed by 57 dogs.

The septic group was composed mainly by mixed-breed dogs (*n* = 14; 24.5%). The breeds involved were: Labrador Retriever (*n* = 6), German Shepherd (*n* = 4), Jack Russell Terrier and Kurzhaar (*n* = 3 each), Dobermann, Golden Retriever, Rottweiler and English Setter (*n* = 2 each). The remaining 19 dogs belonged to other breeds (1 dog each breed). The median age was 7.6 years (4.3–10.7 years). There were 31 females and 26 male dogs. Fourteen dogs out 57 (24.5%) died during the hospitalization and constituted the non-survivor group. The survivor group was then composed by 43 dogs. Subtyping the foci of the septic process, 17 dogs (29.8%) had a gastrointestinal origin of sepsis, 13 (22.8%), and 12 dogs (21%) had reproductive and respiratory sepsis, respectively, 9 dogs had dermatological sepsis as primary site, and urinary and orthopedic sepsis were present in 6 dogs (3 dogs each). No association between sepsis site and mortality was found (*p* = 0.9).

The SIRS group was built using another study population (8). The SIRS group was composed by 57 dogs and they were mainly mixed breed dogs (*n* = 17), followed by Cocker Spaniel and German Shepherd (*n* = 5 each breed), Rottweiler and Dachshund (*n* = 3 each breed), Dobermann, Lagotto Romagnolo, and Springer Spaniel (*n* = 2 each). The remaining 18 dogs belonged to other breeds (1 dog each breed). The median age was 9.5 years (4–11.2 years). In the SIRS group there were 25 females and 25 males. Twenty dogs (35%) of SIRS group died during hospitalization.

### Comparisons Between Septic and SIRS Group in Admission

**Figure 1:**
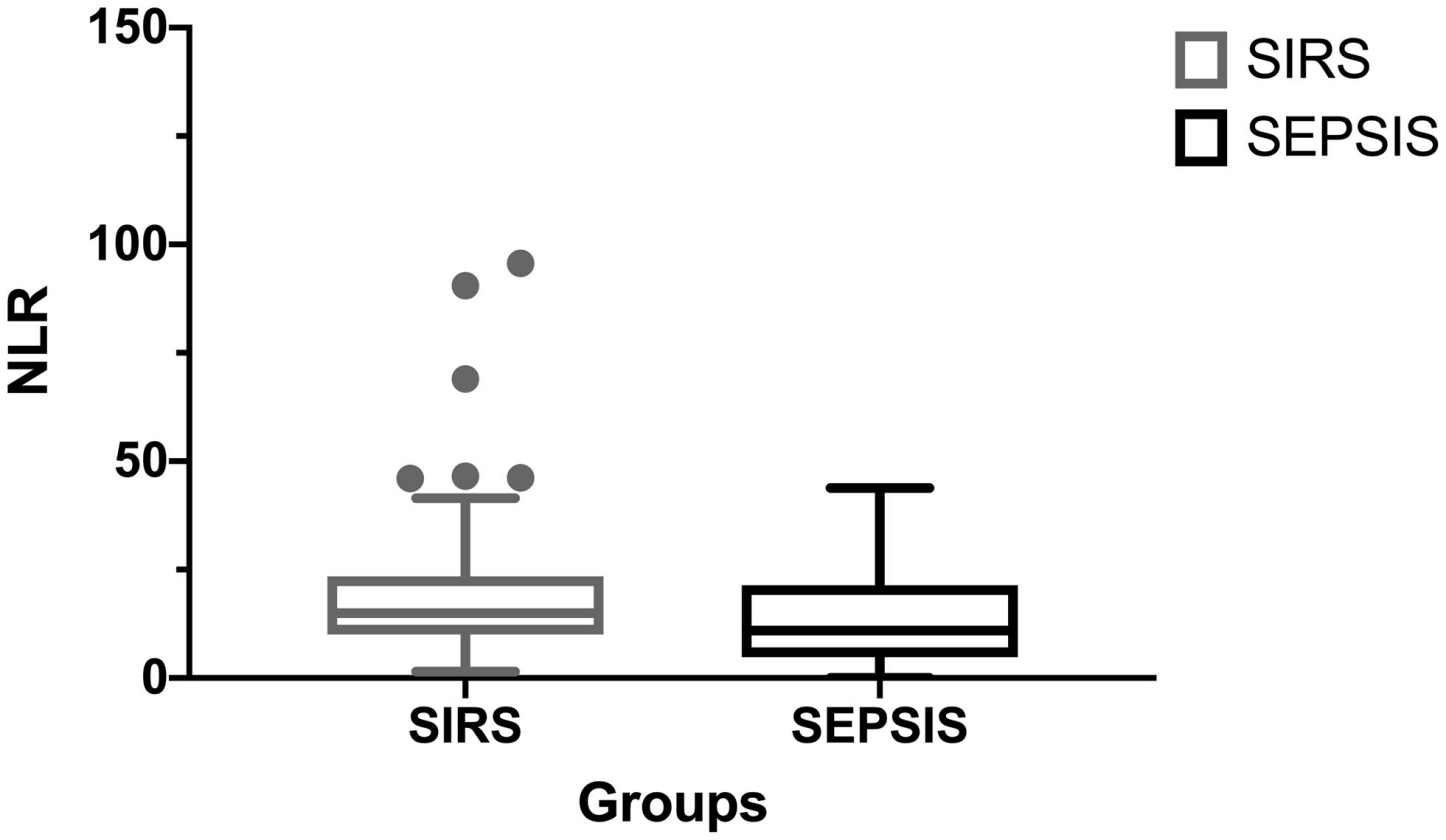
Comparison of NLR between SIRS and septic dogs.

**Figure 2:**
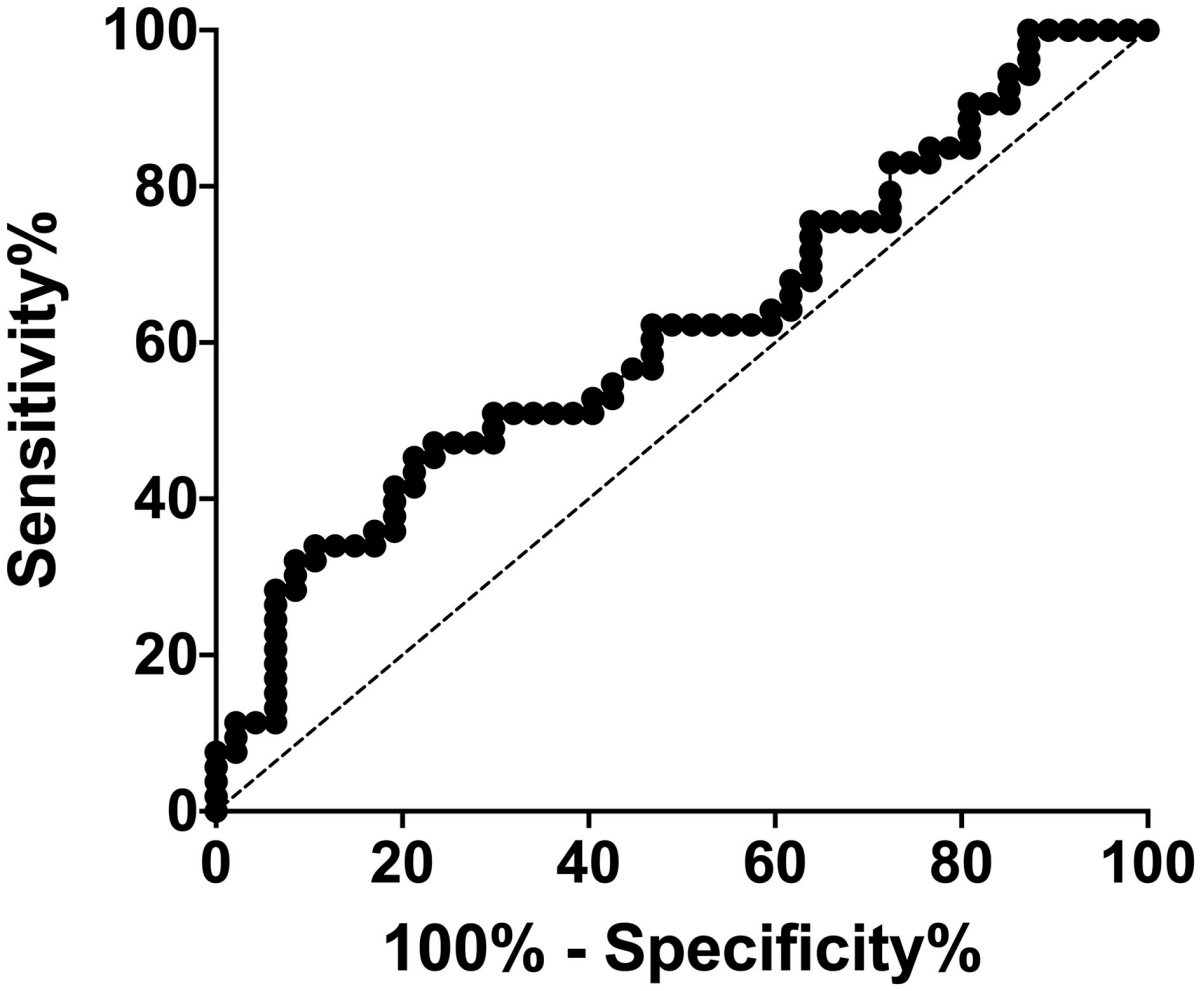
Receiver-operating characteristic curve of NLR.

Results from the univariate analysis between septic and SIRS group are reported in Table 1. Briefly, septic dogs had significant lower median segmented neutrophils count and NLR compared to SIRS dogs (*p* = 0.02 and *p* = 0.04; Figure 1, respectively). All the ROC curves AUCs and respective *p*-values are displayed in Table 2. The ROC curve indicated that NLR could be used to predict the presence of sepsis with a value <8.5 (AUC 0.62, *p* = 0.04, sensitivity 50%, specificity 78%; Figure 2). A value of NLR <5.6 had a specificity of 91% to detect sepsis (sensitivity 32%). Lastly, septic dogs had a higher prevalence of toxic neutrophil than SIRS ones (*p* = 0.01).

**Table 1:** Results from the univariable analysis between septic and SIRS dogs.

**Parameter**	**Reference interval**	**Septic group (*n* = 57)**	**SIRS group (*n* = 57)**	***p*-value**
WBC (K/μL)	5.05–16.7	16.5 (7.9–30.2)	21.06 (12.9–30.2)	0.10
Segmented neutrophils (K/μL)	3.7–11.9	12.0 (5.1–21.1)	17.63 (10–23.4)	**0.02**
Band neutrophils (K/μL)	0	0.4 (0–2.6)	0.42 (0–1)	0.32
Lymphocytes (K/μL)	0.7–5.1	0.92 (0.4–1.8)	0.84 (0.18–1.8)	0.31
Monocytes (K/μL)	0.2–1.7	1.26 (0.56–3)	1.74 (0.9–2.9)	0.44
PLT (K/μL)	148–484	264 (61–361.5)	182 (89.5–281.5)	0.15
MPV (fL)	8.7–13.2	10.3 (8.9–11.6)	9.65 (8.4–10.9)	0.08
Presence of toxic neutrophils	Absent	15/57 (26%)	5/57 (8.7%)	0.01^*^
NLR	NA	10.97 (4.8–21.4)	14.97 (10–23.4)	**0.04**
NBNLR	NA	0.04 (0–0.2)	0.02 (0–0.13)	0.66
PLR	NA	214 (58.4–457.3)	180.2 (52–374.7)	0.58
MLR	NA	1.27 (0.8–2.8)	1.72 (0.7–3)	0.53

*All p-values were obtained using Mann-Whitney U-test and all continuous parameters are reported as median and 25th−75th percentile in brackets. P-values in bold characters resulted statistically significant*.

* *Chi-square test. NA, not applicable (RI for these parameters has not been validated in dogs)*.

**Table 2:** Receiver-operating characteristic curves of continuous variables included in the study.

**Parameter**	**AUC**	***p*-value**
WBC (K/μL)	0.58	0.1
Segmented neutrophils (K/μL)	0.62	0.05
Band neutrophils (K/μL)	0.55	0.32
Lymphocytes (K/μL)	0.55	0.31
Monocytes (K/μL)	0.54	0.44
PLT (K/μL)	0.57	0.14
MPV (fL)	0.61	0.08
NLR	0.62	**0.04**
NBNLR	0.53	0.63
PLR	0.53	0.58
MLR	0.53	0.52

*AUC, area under the curve*.

### May Changes in Leukogram, Platelets, and WBC/PLT Ratios Have Prognostic Significance in Septic Dogs?

Parameters estimates of each GEE model are reported in Table 3. In particular, we found that for a 1-unit increase of PLR and MLR, the risk of death increased by 50.5 and 60%, respectively.

**Table 3:** Parameters estimates in GEE models of septic dogs.

**Parameter**	**Model effects**	**Exp(B) (95% CI)**	***p*-value**
WBC (K/μL)	Time	0.98 (0.15–0.94)	0.73
	WBC	0.99 (0.96–1.02)	0.74
Segmented neutrophils (K/μL)	Time	0.98 (0.85–1.12)	0.98
	Segmented neutrophils	0.99 (0.96–1.03)	0.78
Band neutrophils (K/μL)	Time	0.99 (0.86–1.14)	0.88
	Band neutrophils	1.02 (0.83–1.3)	0.88
Lymphocytes (K/μL)	Time	0.66 (0.42–1.04)	0.07
	Lymphocytes	0.73 (0.36–1.46)	0.38
	Time^*^ Lymphocytes	1.4 (0.93–2)	0.12
Monocytes (K/μL)	Time	0.99 (0.94–1.06)	0.87
	Monocytes	0.98 (0.82–1.18)	0.88
PLT (K/μL)	Time	1.02 (0.91–1.14)	0.73
	PLT	0.99 (0.99–1)	0.75
MPV (fL)	Time	1.01 (0.93–1.1)	0.77
	MPV	1.04 (0.82–1.32)	0.75
NLR	Time	1.03 (0.86–1.23)	0.77
	NLR	1.04 (0.99–1.09)	0.09
NBNLR	Time	1.4 (0.95–1.96)	0.09
	NBNLR	1.3 (0.82–1.97)	0.27
	Time^*^ NBNLR	0.24 (0.04–1.63)	0.14
PLR	Time	2.3 (0.95–5.8)	0.06
	PLR	1.01 (1–1.05)	**0.006**
	Time^*^ PLR	0.997 (0.994–0.999)	**0.009**
MLR	Time	0.90 (0.71–1.16)	0.44
	MLR	1.46 (1.04–2.05)	**0.03**

Each model has as dependent variable the outcome (survivor/non-survivor) and as fixed factor and covariates, time and each parameter. Time

* *X, interaction between time and another variable. P-values in bold characters resulted statistically significant*.

## Discussion

During sepsis, the hematologic system plays a key-role both in the response to a septic injury and resolution of this syndrome (18) Despite our first hypothesis, among conventional leukogram, and platelets parameters, only segmented neutrophils were significantly lower in septic group compared to SIRS dogs. In addition, the prevalence of toxic neutrophils in septic dogs compared to the SIRS ones were also increased. Our results are in line with what reported by Troìa et al. which demonstrated that dogs with more severe septic inflammatory process had lower neutrophil count (5). Neutrophils play important role in the innate immune system, mainly phagocytosing infectious agents (18). Generally, a neutrophilic leukocytosis can be the result of recruitment of mature neutrophils from the marginal into the circulating pool, mobilization of mature neutrophils from the bone marrow, and increased leukopoiesis, which is a typical appropriate response to many types of infections (18). On the contrary, in other cases of sepsis, a neutropenia may occur as the result of depletion of bone marrow precursors or leukocyte migration into the infected focus, overcoming the bone marrow ability to replace them (18). Based on the same mechanisms, during sepsis, signs of neutrophilic toxicity may occur (5, 17, 18). We found a significant higher prevalence of neutrophilic toxicity in septic dogs compared to SIRS group. Toxic change refers to the morphological abnormalities of neutrophils seen in severe inflammatory diseases, especially in those with severe bacterial infection (17, 18). In our study blood smear evaluation were examined or reviewed by a single trained clinical pathologist, less experienced operators may have some difficulties with mild toxic changes in neutrophils. For this reason, in doubtful septic cases, a blood smear review of a well-trained clinical pathologist is recommended.

Interestingly and according to our previous study (8), the NLR resulted significantly lower in septic dogs compared to SIRS group. Firstly, Zahorec et al. proposed the NLR as a marker of human patients with sepsis (19). The NLR has been identified in human medicine as a simple and ready-available biomarker, that was proposed as an independent predictor of mortality both in sepsis, and in other clinical conditions (19, 20). In addition, the NLR seemed to have discriminatory capacity to predict sepsis and differentiate sepsis from SIRS (21). In veterinary medicine, NLR has been analyzed initially in oncology veterinary patients, evaluating its prognostic and diagnostic ability to predict mortality, and the presence of malignances (22–25). More recently, NLR has been investigated also in dogs with septic peritonitis, SIRS dogs and a healthy group of dogs (7). In this latter study, an NLR ≥6 had an 84.39% sensitivity and 86.95% specificity to identify dogs with SIRS, however, was unable to distinguish septic and non-septic dogs (7). In septic dogs, a lower NLR compared to a SIRS dog, may be due to neutropenia, which may occur more commonly in sepsis (8, 17). In addition, in our study a value of NLR <5.6 had a specificity of 91% to detect sepsis. However, due to the low sensitivity (32%) and low AUC, this data should be interpreted with caution and it cannot be used in clinical practice yet and needs further investigations.

The second aim of the present study was to investigate if there were any differences between sepsis outcome groups in leukocytes, platelets and WBC/PLT ratios changes. Based on our results, PLR and MLR were significantly different between survivors and non-survivors overt hospitalization time. During inflammation, increased thrombopoietin and cytokines levels contribute to increased megakaryocytosis, that causes an increased production of PLT and a reduction in their size (26). Based on this consideration, the evaluation of both PLT and PLR in septic dogs might help to explain platelet kinetics, and maybe facilitate early identification of sepsis, being an available predictor of outcome. Platelet and PLT indices have been shown to have prognostic significance in septic people (27). In canine sepsis, both thrombocytopenia or thrombocytosis may occur, although their presence was not related with the mortality (6). However, high MPV seem to be significantly associated with mortality of dogs with septic peritonitis, imparting almost a 3-fold relative risk of death (6). In our population, it seems that PLR can be considered a predictor of non-survival. A possible explanation may be that in non-survivors, the septic process remains ongoing, causing a persistent thrombocytosis, which may be responsible of higher PLR through the time.

In our population, non-survivors seem to have a persistent high or increase MLR compared to survivors. To the best of the authors' knowledge, this is the first report regarding MLR in canine sepsis. Monocytes are an essential component of the innate immune system which may be elevated in various diseases, especially those in which phagocytosis is required (17). Monocytes, generally migrates from blood to various tissues, where they substitute apoptotic macrophages and dendritic cells (17). In particular, when infection induces an inflammatory response, monocytes migrate to inflamed tissues, and differentiate into macrophages killing pathogens *via* phagocytosis, and produce reactive oxygen species and inflammatory cytokines (17). However, human literature on MLR in sepsis is lacking. One study investigated both NLR and MLR in patient groups hospitalized for fever due or not to infection, concluding that both NLR and MLR may be useful tools in the diagnosis of sepsis (28). Our finding may be related to the persistent infection and, thus, to a persistent request of monocytes in the sepsis focus together with tissue necrosis, which may result in a persistent higher MLR in non-survivors septic dogs during hospitalization.

In conclusion, our results showed how, at the hospital admission, the evaluation of NLR results are different between SIRS and septic dogs. However, in our population of dogs NLR showed low sensitivity in differentiating SIRS and septic dogs, thus further studies are warranted to better understand its clinical utility. In addition, the monitoring of some CBC parameters, especially PLR and MLR may be useful in the establishment of septic dogs prognosis.

## Data Availability Statement

The datasets generated for this study are available under reasonable request.

## Ethics Statement

Ethical review and approval was not required for the animal study because retrospective study. Written informed consent was obtained from the owners for the participation of their animals in this study.

## Author Contributions

EG, AP, and VM designed the study, analyzed data, co-wrote, and edited the manuscript. IL and GL edited the manuscript. All authors contributed to read and approved the final manuscript.

## Conflict of Interest

The authors declare that the research was conducted in the absence of any commercial or financial relationships that could be construed as a potential conflict of interest.

## Footnotes

^1^Procyte DX, IDEXX Laboratories, Westbrook, USA.

^2^Aerospray Wescor, Delcon, Milan, Italy.

^3^IBM SPSS Statistics, version 25, IBM Corporation, New York, New York, USA.

^4^GraphPad Prism, version 7.0a, GraphPad Software Inc, San Diego, California, USA.
